# The Association of Angiotensin Converting Enzyme and Angiotensinogen Gene Polymorphism With Dilated Cardiomyopathy: A Systematic Review and Meta-Analysis

**DOI:** 10.31083/RCM39763

**Published:** 2025-10-21

**Authors:** Songbai Du, Ningting Jiang, Yilin He, Zhiming Li, Chang Liu, Rong Luo

**Affiliations:** ^1^Department of Cardiology, Nanbu County People's Hospital, 637300 Nanchong, Sichuan, China; ^2^Institute of Geriatric Cardiovascular Disease, Chengdu Medical College, 610500 Chengdu, Sichuan, China

**Keywords:** dilated cardiomyopathy, gene polymorphism, angiotensin converting enzyme, angiotensinogen, meta-analysis

## Abstract

**Background:**

Limited evidence exists for an association between dilated cardiomyopathy (DCM) and the angiotensin-converting enzyme (*ACE*) gene with an insertion/deletion (*I/D*) angiotensinogen (*AGT*) *M235T* gene polymorphism. A systematic review and meta-analysis were conducted to elucidate the role of *ACE I/D* and *AGT M235T *in the morbidity of DCM. This meta-analysis was performed following the Preferred Reporting Items for Systematic Reviews and Meta-analyses (PRISMA) 2020 guidelines for Abstracts.

**Methods:**

The PubMed, Embase, and Cochrane Library databases, as well as the Chinese Biomedical Literature Database, were reviewed to identify and collect all relevant studies. The association between *ACE I/D*, *AGT M235T* gene polymorphism, and DCM was estimated by pooling the odds ratio (OR) using the RevMan5.4.1 and Stata12.0 software.

**Results:**

A total of 27 eligible studies that explored the* ACE I/D* gene polymorphism in a healthy control group and the DCM patients were included in the present meta-analysis. A recessive genetic model was presented in the *ACE I/D* genotype. The pooled OR (*DD *vs.* DI+II*) following recessive genetic modelling was 1.37 (95% confidence interval (CI): 1.13, 1.66; *p* < 0.01). DCM patients tend to carry the *DD* genotype, indicating that the *ACE I/D* gene polymorphism might be associated with DCM. Similarly, seven studies were analyzed that presented a correlation between *AGT M235T* polymorphism and DCM morbidity. The OR (*MT* *+* *TT* vs. *MM*) value, according to a dominant genetic model, was 1.83 (95% CI: 0.90, 3.73; *p* > 0.05).

**Conclusion:**

The *AGT M235T* polymorphism was not significantly associated with DCM; however, the* ACE I/D* polymorphism was related to a risk of DCM.

## 1. Introduction

Dilated cardiomyopathy (DCM) is a myocardial disease characterized by the dilatation of either the left or both ventricles with impaired systolic function [1]. Despite recent advances in medical and surgical therapies, it remains an important cause of mortality and is a leading indication for heart transplantation. The prevalence of idiopathic DCM is approximately 1 in 250 individuals [2]. However, the causes of dilated cardiomyopathy are heterogeneous and unclear [3]. Nonfamilial DCM may have multifactorial etiologies resulting from an interaction between genetic and environmental factors. Several modifier genes also influence the DCM phenotype. Polymorphisms in the genes involved in the renin-angiotensin system (RAS) are associated with a higher risk of DCM [4, 5, 6].

In the RAS, angiotensinogen (AGT) is synthesized primarily by the liver and released into the blood, where it is cleaved by renin to generate angiotensin I. The latter is subsequently converted to angiotensin II by the angiotensin-converting enzyme (ACE). Angiotensin II is involved in cellular hypertrophy and proliferation [7], and thus regulates cardiac function, blood pressure, and electrolyte homeostasis [8]. The *ACE* gene is located on chromosome 17q23. An insertion/deletion (*I/D*) polymorphism (a 287-base-pair *Alu* repeat sequence) is usually present within intron 16 [9]. The *AGT* gene, located on chromosome 1q4, has an *M235T* polymorphism [10]. The *ACE I/D* gene and/or *AGT M235T* polymorphism are involved in cardiomyopathies [4, 5, 6, 11, 12, 13, 14, 15, 16, 17]. However, some studies could not establish any correlation between *ACE I/D* or the *AGT M235T* genotype and DCM [18, 19, 20, 21, 22, 23, 24, 25, 26, 27, 28, 29, 30, 31, 32, 33, 34, 35, 36]. Thus, the role of *ACE I/D* and *AGT M235T* genotype in the pathogenesis of nonfamilial DCM remains controversial. We decided to perform a meta-analysis to evaluate the effects of *ACE I/D* and *AGT M235T* gene polymorphism on the DCM phenotype. We reviewed case-control studies which explored the *ACE I/D* gene (OMIM number: 106180) and *AGT M235T* (OMIM number: *106150) gene polymorphism in healthy control and DCM patients, to determine the role of these two gene polymorphisms.

## 2. Methods

### 2.1 Search Strategy

A comprehensive search for relevant studies was conducted in PubMed, Embase, Cochrane Library, and the China Biology Medicine disc till February 2025. The review was prepared on the basis of published protocols [37, 38]. To find studies exploring the relationship between *ACE I/D* with DCM, the search words used in the PubMed database were: ACE or “angiotensin converting enzyme”, polymorphism or mutation, and “dilated cardiomyopathy or dilated cardiomyopathies”. Alternatively, ACE was replaced with “angiotensinogen” or “AGT” for studies related to angiotensinogen *M235T* genotype and DCM. Details of the Embase search strategy are described in **Supplementary Material I**. Language was not a limiting factor in our search.

### 2.2 Study Selection

Two authors independently reviewed all studies and collected the data using a standard information extraction approach following the Preferred Reporting Items for Systematic Reviews and Meta-analyses (PRISMA) statement [39, 40]. The studies included met with the following criteria: (1) a cohort study highlighting the *ACE I/D* allele polymorphism or *AGT M235T* gene polymorphism; (2) the case group involved DCM patients. (3) A healthy control group was included in all studies. The exclusion criteria were as follows: (1) reviews, comments, case reports, meta-analysis and animal experiments; (2) other studies in which neither *ACE* nor *AGT* gene polymorphism was explored in DCM or a control group in a DCM or control group.

### 2.3 Data Extraction and Risk of Bias

The following data were collected: the first author of the studies, year of publication, genotypes of patients and controls, *p*-values to calculate Hardy-Weinberg equilibrium (HWE) in the control group, the source of control subjects, and diagnosis methods or criteria in DCM patients.

The quality of studies was independently assessed by two authors using a revised bias assessment score (**Supplementary Material II**) [41]. Total scores ranged from 0 (worst) to 13 (best). Any dissension was resolved by discussion.

### 2.4 Statistical Analysis

All data for statistical analysis were obtained from the published paper or meeting abstracts. RevMan Software 5.4.1 (Cochrane Collaboration, https://www.cochrane.org/products-and-services/review-writing-software) was used for pooling the odds ratio (OR) in the meta-analysis. Meta-regression and calculation of genetic models were performed with Stata 12.0 software (StataCorp LP, College Station, TX, USA). The most appropriate genetic models were calculated following protocols described previously [41, 42]. Continuity correction by adding 1 into the 0 genotype was applied. For *ACE I/D* gene polymorphism, a recessive genetic model was used. For the role of *AGT M235T* genotype in DCM, a dominant model was used. Meta-regression was used to explore potential sources of heterogeneity. A *p*-value less than 0.10 and I^2^ greater than 50% were considered to be significant for statistical heterogeneity. The random-effect model was used in the analysis [43, 44]. Sensitivity analysis was also performed to test the robustness of the results by excluding studies that deviated from HWE. In addition, a subgroup analysis to determine the origin of the patients was also performed. Begg’s test, Egger’s test and funnel plots were used to assess and avoid any publication bias.

## 3. Results

### 3.1 Search Results

**Fig. 1.**
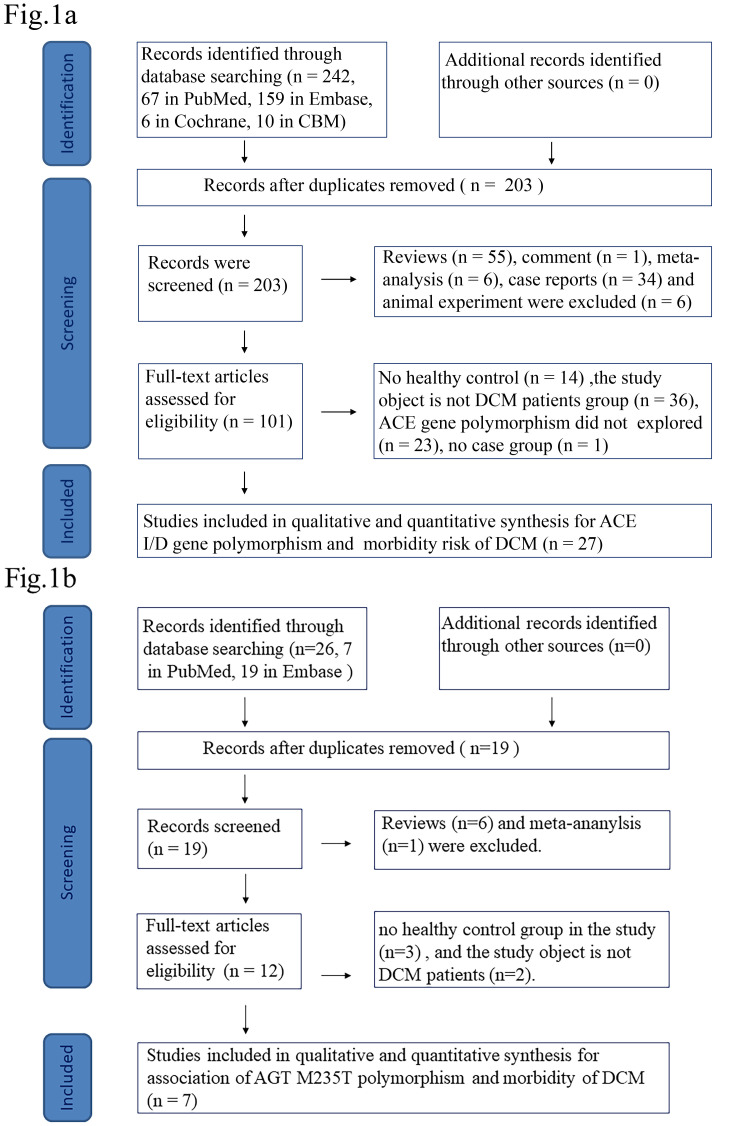
**Flow diagram highlighting the criteria for selection of the studies**. (a) *ACE I/D* flow diagram. (b) *AGT* flow diagram. DCM, dilated cardiomyopathy; ACE, angiotensin converting enzyme; AGT, angiotensinogen; I/D, insertion/deletion; CBM, the China Biology Medicine disc.

A total of 242 studies were retrieved for *ACE* gene polymorphism and 26 studies for *AGT* gene polymorphism from the databases. Among them, 29 studies were included in the analysis. Of these, 27 studies [4, 5, 6, 11, 12, 13, 14, 16, 17, 18, 19, 20, 21, 22, 23, 24, 25, 27, 28, 29, 30, 31, 32, 33, 34, 35, 36] were included in the meta-analysis to show an association between *ACE I/D* gene polymorphism and DCM (Fig. 1a); 7 studies [11, 15, 19, 20, 26, 28, 30] correlated *AGT M235T* gene polymorphism and DCM (Fig. 1b). Among these, five studies [11, 19, 20, 28, 30] were included in both the *ACE I/D* and *AGT M235T* gene polymorphism analysis. Fig. 1 shows the flow diagram of the criteria used in the study selection. Table 1a (Ref. [4, 5, 6, 11, 12, 13, 14, 16, 17, 18, 19, 20, 21, 22, 23, 24, 25, 27, 28, 29, 30, 31, 32, 33, 34, 35, 36]) and Table 1b (Ref. [11, 15, 19, 20, 26, 28, 30]) list the selected studies and the main characteristics in the control and DCM Group. The quality of the selected studies is shown in **Supplementary Material III**.

**Table 1a.** **Characteristics of eligible studies associating *ACE I/D* gene polymorphism and DCM**.

First author, Year	Ethnic	DCM				Control				HWE in control *p* value	Control subjects	Diagnosis methods/criteria in DCM patients
		Genotypes				Genotypes						
		N	II	ID	DD	N	II	ID	DD			
Kose *et al*. 2014 [22]	Turkey	36	8	17	11	104	16	47	41	0.6780	Healthy subjects	Echocardiography
Kong *et al*. 2012 [34]	Han Chinese	101	21	48	32	105	30	53	22	0.8744	Healthy individuals	WHO/ISFC diagnostic criteria of DCM in 1995
Mahjoub *et al*. 2010 [5]	Tunisia	76	12	38	26	151	46	83	22	0.1162	Age, sex and ethnicity matched controls without any previous history of cardiovascular disorders	Criteria provided by the World Health Organization.
Shan *et al*. 2001 [18]	Chinese	83	27	25	31	155	50	80	25	0.4564	Healthy individuals	WHO/ISFC diagnostic criteria of DCM in 1995, echocardiography
Zou *et al*. 2003 [17]	Chinese	43	12	18	13	53	28	20	5	0.6095	Age and gender matched healthy individual and blood donor	WHO/ISFC diagnostic criteria of DCM in 1995, echocardiography
Wu *et al*. 2002 [35]	Chinese	43	14	22	7	63	23	28	12	0.5092	Healthy individuals	WHO/ISFC diagnostic criteria of DCM in 1995, echocardiography
Küçükarabaci *et al*. 2008 [23]	Turkey	29	5	18	6	20	7	9	4	0.7229	Healthy subjects	Echocardiography
Rai *et al*. 2008 [6]	India	51	8	33	10	164	47	87	30	0.3532	Healthy, age, sex, and ethnicity matched controls without any previous history of cardiovascular disorders	Echocardiography
Jurkovicova *et al*. 2007 [19]	Caucasian population of Slovakia	110	21	50	39	156	38	78	40	0.9984	Healthy control subjects matched to patients by gender and age	Not defined
Covolo *et al*. 2003 [36]	Italy	122	17	62	43	230	39	105	86	0.4744	Born in Italy, had no clinical symptoms or signs suggesting the presence of HF, and no history of CHD or IDC	Echocardiography
Tiago *et al*. 2002 [20]	South Africa	157	26	60	71	225	18	105	102	0.2050	Healthy, unrelated Black South Africans were recruited from the general population of surrounding districts	Echocardiography
Tiret *et al*. 2000 [28]	France	422	94	200	128	387	71	190	126	0.9662	Age matched French population without clinical history of cardiovascular disease or insulindependent diabetes	Radionucleotide angiography or echocardiography
Straburzynska-Migaj 2005 [32]	Poland	52	14	19	19	110	28	48	34	0.1910	Healthy pregnant women	Echocardiography
Candy *et al*. 1999 [21]	Black South Africans	171	27	72	72	106	13	46	47	0.7376	Age matched, unrelated black South Africans free of cardiovascular disease	Echocardiography and radionuclide ventriculography
Vancura *et al*. 1999 [29]	Czech	90	27	33	30	287	70	146	71	0.7677	Residents from 1 district in central Bohemia	Not defined
Yamada *et al*. 1997 [30]	Japanese	88	36	35	17	122	50	55	17	0.7640	Healthy individuals	Echocardiography, coronary angiography and left ventriculography
Sanderson *et al*. 1996 [27]	Chinese	51	20	25	6	183	71	88	24	0.6882	Healthy subjects and patients without heart disease	The criteria set by the World Health Organization, Echocardiography, and cardiac catheterization
Montgomery *et al*. 1995 [25]	United Kingdom	99	18	50	31	364	84	168	112	0.1729	Local general practice group	The criteria recommended by the WorldHealth Organization, Echocardiography
Raynolds *et al*. 1993 [14]	USA	112	22	50	40	89	20	50	19	0.2431	Actual or prospective heart donors and healthy volunteers with normal ECG and echocardiographic studies	Echocardiogram
Ozhan *et al*. 2004 [31]	Turkey	35	4	17	14	88	11	28	49	0.0411	Healthy unrelated age-and sex-matched subjects	Transthoracic echocardiogram
Kurbanov *et al*. 2014 [24]	Uzbekistan	102	33	45	24	60	34	14	12	0.0004	Healthy subjects	The diagnostic criteria for DCM (WHO, 1995), echocardiography
Harn *et al*. 1995 [4]	Chinese	35	2	13	20	35	2	24	9	0.0112	Patients with normal donor-screening echocardiograms and normal coronary arteriograms	Echocardiography
Rani *et al*. 2017 [11]	India	177	15	120	42	200	72	86	42	0.0891	Healthy, ethnicity-matched unrelated subjects without any family history of heart disease, hypertension, diabetes or any other chronic ailments	Echocardiography
Schmidt *et al*. 1996 [33]	Austria	14	4	7	3	95	21	38	36	0.0801	Healthy control group	Ultrasonography
Chen *et al*. 2017 [16]	Chinese	64	17	29	18	120	51	57	12	0.4957	Healthy individuals	Diagnostic criteria of Chinese Society of Cardiology
Goncalvesova *et al*. 2005 [12]	Slovak	70	15	29	26	103	28	51	24	0.9336	General Slovak population	Echocardiography
Berg *et al*. 2012 [13]	Bashkortostan	27	10	9	8	82	32	41	9	0.4394	Healthy people, age, gender and ethnicity matched, without chronic diseases as well as without pathology of cardiovascular system in the anamnesis	WHO classification criteria, echocardiogram, and coronarography

ACE, angiotensin-converting enzyme; I/D, insertion/deletion; DCM, dilated cardiomyopathy; HWE, Hardy-Weinberg equilibrium; WHO/ISFC, World Health Organization/International Society of Forensic Genetics; CHD, coronary Heart Disease; IDC, idiopathic dilated cardiomyopathy; ECG, electrocardiograph.

**Table 1b.** **Characteristics of eligible studies correlating *AGT M235T* gene polymorphism with DCM**.

First author, Year	Ethnic	DCM				Control				HWE in control *p* value	Control subjects	Diagnosis methods in DCM patients
		Genotypes				Genotypes						
		N	MM	MT	TT	N	MM	MT	TT			
Jurkovicova *et al*. 2007 [19]	Caucasian population of Slovakia	110	31	51	28	156	62	69	25	0.4339	Healthy control subjects matched to patients by gender and age	Not defined
Tiago *et al*. 2002 [20]	South Africa	157	0	55	102	225	0	58	167	0.0265	Healthy, unrelated Black South Africans were recruited from the general population of surrounding districts	Echocardiography
Tiret *et al*. 2000 [28]	France	428	157	200	71	398	131	195	72	0.9695	Age matched French population without clinical history of cardiovascular disease or insulin-dependent diabetes	Radionucleotide angiography or echocardiography
Pávková Goldbergová *et al*. 2011 [26]	Czech Republic	91	23	55	13	203	65	101	37	0.8377	Not define	Not defined
Yamada *et al*. 1997 [30]	Japan	88	3	29	56	122	2	44	76	0.1190	Healthy individuals	Echocardiography, left ventriculography, and coronary angiography
Rani *et al*. 2017 [11]	India	177	15	120	42	200	72	86	42	0.0891	Healthy, ethnicity-matched unrelated subjects without any family history of heart disease, hypertension, diabetes or any other chronic ailments	Echocardiography
Ullah *et al*. 2019 [15]	Pakistan	35	20	0	15	42	38	0	4	0.0000	Ethnically matched healthy controls without any history for heart abnormality, hypertension and diabetes	Not defined

DCM, dilated cardiomyopathy; HWE, Hardy-Weinberg equilibrium; AGT, angiotensinogen.

### 3.2 HWE and the Minor Allele Frequency

Calculated HWE values in the control group are shown in Table 1a and Table 1b. Table 2a (Ref. [4, 5, 6, 11, 12, 13, 14, 16, 17, 18, 19, 20, 21, 22, 23, 24, 25, 27, 28, 29, 30, 31, 32, 33, 34, 35, 36]) and Table 2b (Ref. [11, 15, 19, 20, 26, 28, 30]) represent the *ACE I/D* and *AGT M236T* gene polymorphism (minor allele). In the studies associating *ACE I/D* gene polymorphism with DCM, the minor allele in the control group (*D* allele) had an allele frequency of 48.86% (95% CI: 44.25%, 53.47%). The minor allele in the control group for the *AGT* genotype was the T allele with a frequency of 49.04% (95% CI: 24.49%, 73.59%).

**Table 2a.** **Estimation of the minor allele (*D*) frequency in control groups for *ACE I/D* genotype**.

First Author, Year	D allele frequency	Total frequency	Allele D allele percent (%)
Kong *et al*. 2012 [34]	97	210	46.1905
Shan *et al*. 2001 [18]	130	310	41.9355
Zou *et al*. 2003 [17]	30	106	28.3019
Wu *et al*. 2002 [35]	52	126	41.2698
Kose *et al*. 2014 [22]	129	208	62.0192
Mahjoub *et al*. 2010 [5]	127	302	42.0530
Küçükarabaci *et al*. 2008 [23]	17	40	42.5000
Rai *et al*. 2008 [6]	147	328	44.8171
Jurkovicova *et al*. 2007 [19]	158	312	50.6410
Covolo *et al*. 2003 [36]	277	460	60.2174
Tiago *et al*. 2002 [20]	309	450	68.6667
Tiret *et al*. 2000 [28]	442	774	57.1059
Straburzynska-Migaj *et al*. 2005 [32]	116	220	52.7273
Candy *et al*. 1999 [21]	140	212	66.0377
Vancura *et al*. 1999 [29]	288	574	50.1742
Yamada *et al*. 1997 [30]	89	244	36.4754
Sanderson *et al*. 1996 [27]	136	366	37.1585
Montgomery *et al*. 1995 [25]	392	728	53.8462
Raynolds *et al*. 1993 [14]	88	178	49.4382
Ozhan *et al*. 2004 [31]	126	176	71.5909
Kurbanov *et al*. 2014 [24]	38	120	31.6667
Harn *et al*. 1995 [4]	42	70	60.0000
Rani *et al*. 2017 [11]	170	400	42.5000
Schmidt *et al*. 1996 [33]	110	190	57.8947
Chen *et al*. 2017 [16]	81	240	33.7500
Goncalvesova *et al*. 2005 [12]	99	206	45.0583
Berg *et al*. 2012 [13]	59	164	35.9756

Note, Pooled D allele prevalence (%): 48.63 (95% CI: 44.05, 53.20). ACE, angiotensin converting enzyme; I/D, insertion/deletion.

**Table 2b.** **Estimation of the minor allele (*T*) frequency in control groups for *AGT M235T* genotype**.

First Author, Year	T allele frequency	Total frequency	Allele T allele percent (%)
Jurkovicova *et al*. 2007 [19]	119	312	38.1410
Tiago *et al*. 2002 [20]	392	450	87.1111
Tiret *et al*. 2000 [28]	339	796	42.5879
Pávková Goldbergová *et al*. 2011 [26]	175	406	43.1034
Yamada *et al*. 1997 [30]	196	244	80.3279
Rani *et al*. 2017 [11]	170	400	42.5000
Ullah *et al*. 2019 [15]	8	84	9.5238

Note, Pooled T allele prevalence (%): 49.044 (95% CI: 24.49, 73.59). AGT, angiotensinogen.

### 3.3 Meta-Analysis of the Association Between Genotype and DCM Phenotype

**Fig. 2.**
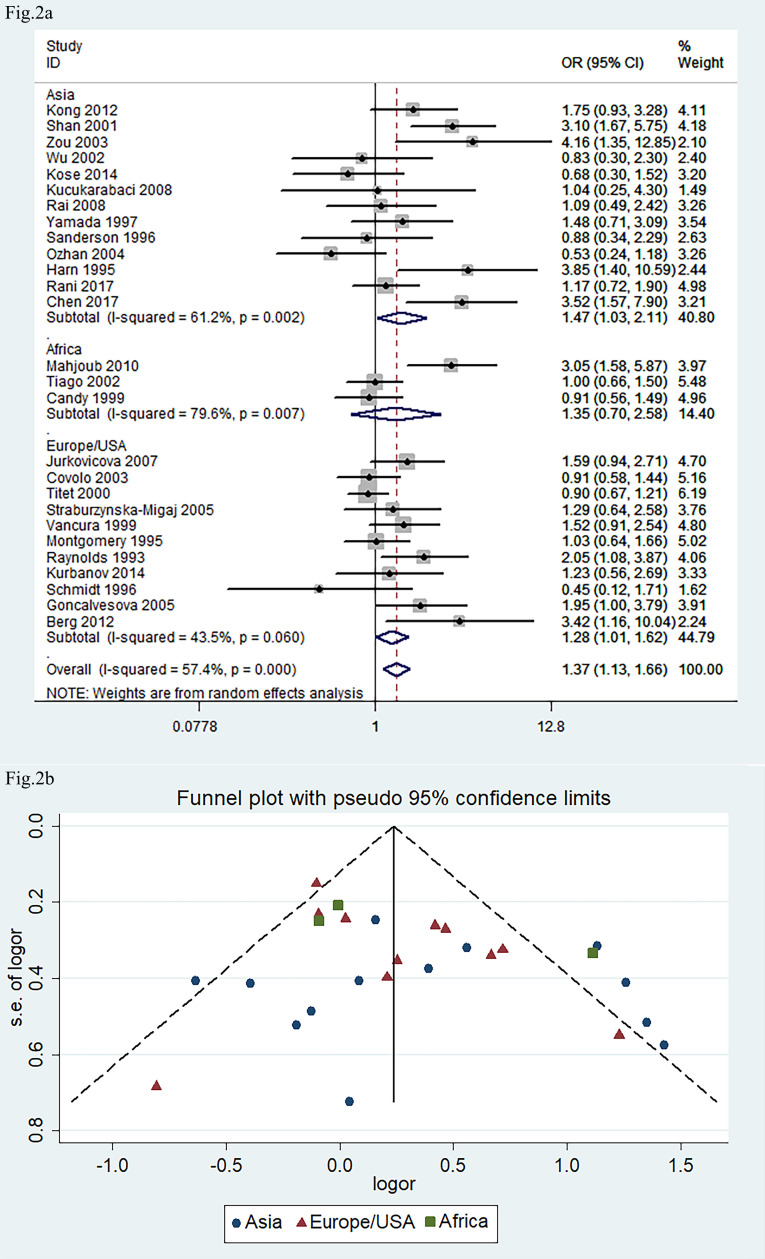
**Forest plot and funnel plot of meta-analysis about *ACE I/D* gene polymorphism in association with risk of DCM phenotype**. (a) Forest plot. (b) Funnel plot. The pooled OR in (a) indicated the OR of *DD* vs. *DI+II* genotypes. The pooled OR was 1.37 (95% CI: 1.13, 1.66; *p* < 0.01). The Begg’s test and Egger’s test indicated that there was no obvious publication bias. ACE, angiotensin converting enzyme; I/D, insertion/deletion; DCM, dilated cardiomyopathy.

The 27 eligible studies, connecting *ACE* I/D allele polymorphism with the risk of DCM, included 2460 medical cases and 3857 healthy subjects as the control for the meta-analysis. The recessive genetic model was selected for the case-control studies, in which the comparison of *DD* vs. *DI+II* was made. The pooled OR as per the regressive genetic model was 1.37 with the random-effect model (95% CI: 1.13, 1.66; *p* < 0.01, I^2^ = 57%; Fig. 2a). These results suggest that the frequency of the *DD* genotype was higher in DCM patients than that seen in the control group. After excluding three studies that had deviated from HWE [4, 24, 31], OR (*DD* vs. *DI+II*) was found to be 1.38 (95% CI: 1.14, 1.68, *p* < 0.01, I^2^ = 56%, **Supplementary Fig. 1**. Sensitivity analysis indicated that the statistical result did not vary even after excluding any single study. Since the quality scores in studies published before 2000 might be different compared to post-2010 studies, reflecting advancements in genotyping and study design, we performed the meta-analysis with the random-effect model after excluding the older studies before 2000, and the pooled OR is 1.41 (95% CI: 1.10, 1.80, **Supplementary Fig. 2**). The pooled OR is 1.32 (95% CI: 1.07, 1.65) if the studies after 2010 are excluded (**Supplementary Fig. 3**). These results indicate that the results of the meta-analysis are robust. Sub-group analysis revealed that the pooled OR (*DD* vs. *DI*+*II*) was statistically significant in the Asian and in the European/USA population (*p* < 0.05) but not in the African population. Publication bias was verified by Begg’s test and Egger’s test (*p* > 0.05; Fig. 2b). Meta-regression analysis indicated that neither the time of publication nor the origin of the population significantly contributed to the heterogeneity in *ACE I/D* gene polymorphism (*p* > 0.05).

The seven eligible studies, associating *AGT M235T* gene polymorphism with DCM, included 1086 DCM patients and 1346 healthy controls. A dominant model (genetic model) was selected, and the comparison of *MT+TT* vs. *MM* was made for the meta-analysis.

**Fig. 3.**
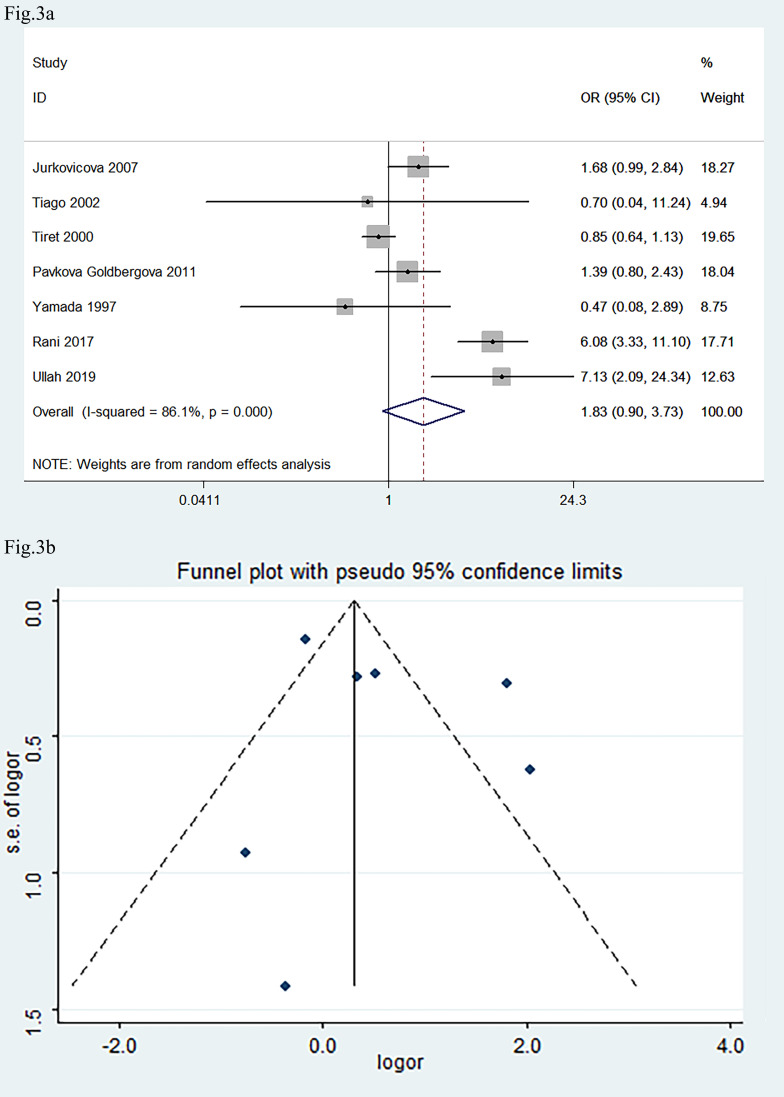
**Forest plot and funnel plot of the meta-analysis correlating *AGT M235T* gene polymorphism with the development of DCM**. (a) Forest plot. (b) Funnel plot. OR in (a) indicated the OR of *MT+TT* vs. *MM* genotypes. The pooled OR was 1.83 (95% CI: 0.90, 3.73; *p* > 0.05). The Begg’s test and Egger’s test indicated that there was no significant publication bias. AGT, angiotensinogen; DCM, dilated cardiomyopathy.

The pooled OR was 1.83 (Fig. 3a: 95% CI: 0.90, 3.73; *p* > 0.05, I^2^ = 86.1%, Fig. 3a), indicating that *AGT* M235T gene polymorphism is not significantly attributed to DCM. Sensitivity analysis indicated that the exclusion of any study did not significantly change the statistical result. The pooled OR did not significantly change after excluding the studies that did not follow HWE [15, 20] (OR = 1.58, 95% CI: 0.74, 3.37, *p* > 0.05, **Supplementary Fig. 4**). These results indicated that the pooled OR value is credible. Egger’s test and Begg’s test (*p* > 0.05; Fig. 3b) indicated that there is no significant publication bias. Meta-regression analysis indicated that neither time of publication nor origin of the population was the main source of heterogeneity (*p* > 0.05).

## 4. Discussion

Our meta-analysis revealed that *ACE DD* genotype frequency was higher in DCM patients, indicating that *ACE I/D* gene polymorphism might be associated with the risk of DCM. The subgroup analysis indicated that *DD* genotype frequency is higher in the Asian and European/USA population. However, it is not significant in Africans. There are just three studies from the African population. Therefore, this lack of association may be due to the small number of studies involving African populations, which limits the statistical power.

DCM is a disease of unknown etiology characterized by ventricular dilation and impaired systolic function [3]. It clinically manifests in heart pump failure or sudden death [45] and is a major indication for heart transplantation [19].

Mutations in genes encoding sarcomeric structural proteins are known contributors to DCM [46]. However, clinical evaluation of families with DCM often reveals the absence of disease in individuals carrying these mutations [2].

The number of rare variants implicated in DCM in the Exome Variant Server (EVS) database was at least double than reported in genetic studies [2]. In addition, the extent of genetic defects varies even among people with the same mutation within the same family. A fixed predictable genotype-phenotype correlation for a specific mutation has not been reported [3]. It has been proposed that clinical heterogeneity in DCM patients is a result of multiple factors, including age, disease-causing gene mutations, environmental effects, and genetic modifiers [47].

Several genes, including those encoding the components of the RAS, are considered potential modifiers in DCM [5]. RAS is a major regulator of cardiovascular and renal functions, including sodium extraction/reabsorption and water balance [26]. Thus, the systemic or local cardiovascular RAS system contributes to the pathophysiology of various cardiovascular diseases and may play an autocrine or paracrine role in cardiac remodeling and fibrosis [48, 49].

In RAS, renin cleaves a terminal decapeptide from angiotensinogen to form angiotensin I [50], which is further catalyzed (enzymatic removal of a dipeptide) into angiotensin II by ACE. ACE is present on the surface of vascular endothelial cells as a membrane-bound enzyme and circulates in plasma. Cloning of the *ACE* gene revealed a 287 base pair (bp) Alu repeat sequence with an *I/D* polymorphism in intron 16 resulting in three genotypes: *II*, *ID*, and *DD* [51, 52]. This polymorphism was strongly associated with increased expression of ACE and high levels of angiotensin II. The mean plasma ACE level in individuals with the *DD* genotype was almost double that of the II genotype, while subjects with *ID* genotype had intermediate levels [9]. The modulating effect of the *DD* genotype on DCM is due to increased ACE activity [6].

Tan and coworkers [53] reported that both endogenous and exogenous angiotensin II lead to myocyte necrosis, abnormal sarcolem permeability, myocytolysis, fibroblast proliferation, and subsequent replacement fibrosis *in vivo*. In addition, angiotensin II stimulation in cardiac fibroblasts of adult rats *in vitro* results in a higher synthesis of extracellular matrix proteins [54]. This increased extracellular matrix synthesis is a key feature of cardiac fibrosis, a condition where the heart tissue becomes stiff and less elastic [55]. Subsequent myocardial remodeling and increased arterial stiffness may result due to the reduction in left ventricular ejection fraction [6]. Elevated angiotensin II levels are associated with an increased mortality rate in heart failure patients [56].

Cardiac collagen deposition in rats may be regulated by RAS activity [57], and this accumulation can be prevented by non-hypotensive doses of ACE inhibitors. Candy and coworkers [21] asserted that the *D* allele is associated with worsening of left ventricular (LV) systolic function as well as an increase in left ventricular cavity size that occurs in idiopathic DCM. *DD* genotype is an independent predictor of higher mortality, LV systolic performance, as well as cavity size in idiopathic DCM [21]. Clinical trials have underscored the therapeutic importance of ACE inhibitors in heart failure [58]. Experimental data in animals and preliminary studies in humans have demonstrated that early administration of captopril, an ACE inhibitor, attenuated progressive LV dilatation [59].

In the present study, we did not confirm an association between the *AGT M235T* polymorphism and DCM. It is reported that the *AGT* haplotype, which carries the A (-6) G variation in the promoter, and *M235T *polymorphism, is associated with higher plasma AGT levels [26, 60]. Bloem and colleagues [61] also found that the T235 allele frequency was higher in black compared to white children, which correlated with the 19% higher mean angiotensin levels in blacks than in whites. Polymorphism of the *AGT* gene is thus race-specific. It is reported that there was almost complete linkage disequilibrium of G (-6) A with the *M235T* o*f AGT* gene [26]. The null finding for *AGT M235T* may reflect low statistical power (small sample size, n = 7 studies, among them, one study did not take part in the pool OR due to the number of *MM* genotype was zero in both the control and DCM group) or population-specific linkage disequilibrium (e.g., *AGT* haplotypes with promoter). Future studies should prioritize haplotype analysis and larger sample sizes for AGT-related endpoints.

Our result is distinct from a previous genome-wide association study (GWAS) on DCM as we have identified an association between *ACE I/D* single nucleotide polymorphism and DCM [62]. To our knowledge, the criterion of assessing statistical significance in GWAS is stricter than in general comparative studies.

## 5. Limitations

There are some limitations in our meta-analysis. First, the studies included are smaller, especially studies associating *AGT M235T* with DCM (only 7 studies), and *ACE I/D* gene polymorphism in Africa, which may lack statistical power to detect true associations. Second, several studies in this meta-analysis reported only a few patient cases. Finally, the qualities of some studies were not satisfactory; for example, three studies deviated from HWE for *ACE I/D* gene polymorphism. In addition, DCM diagnosis across studies used WHO criteria, echocardiography, or a combination, which may introduce heterogeneity. Standardizing diagnostic thresholds (e.g., left ventricular ejection fraction cutoffs) in future could improve consistency. In the end, the review was not registered. All of these limitations may have affected the results of the present study. Further investigations are required to explain the effect of *AGT M235T* and *ACE I/D* polymorphisms in the pathogenesis of DCM.

## 6. Conclusion

In conclusion, despite the above limitations, the present study has suggested that *ACE I/D*, but not *AGT M235T* gene polymorphism, might be a risk factor for DCM. Additional large-scaled and more rigorous case-control studies are needed to further confirm the role of *ACE I/D* and *AGT M235T* polymorphisms in DCM.

## Data Availability

Data involved in statistics are presented in Table 1a and Table 1b. For further information, please contact the corresponding author.

## Abbreviations

DCM, dilated cardiomyopathy; ACE, angiotensin converting enzyme; AGT, angiotensinogen; RAS, renin-angiotensin system; HCM, hypertrophic cardiomyopathy; EVS database, Exome Variant Server database.

## Supplementary Material

Supplementary material associated with this article can be found, in the online version, at https://doi.org/10.31083/RCM39763.
