# Supplementary material for: The Association of Angiotensin Converting Enzyme and Angiotensinogen Gene Polymorphism With Dilated Cardiomyopathy: A Systematic Review and Meta-Analysis
Source: Rev Cardiovasc Med. 2025 Oct 21;26(10):39763. doi: 10.31083/RCM39763 (PMC12593733; doi:10.31083/RCM39763)
Supplement: Supplementary file 1 [file 2153-8174-26-10-39763-s1.zip › Supplementary material.docx]

Supplementary material Ia

Search strategy used in Embase for ACE I/D genotype:

1. “dilated cardiomyopathy”
2. “dilated cardiomyopathies”
3. 1 or 2
4. polymorphism
5. mutation
6. 4 or 5
7. ACE
8. “angiotensin converting enzyme”
9. 7 or 8
10. 3 and 6 and 9

Supplementary material Ib

Search strategy used in Embase for AGT M235T genotype:

1. “dilated cardiomyopathy”
2. “dilated cardiomyopathies”
3. 1 or 2
4. Polymorphism
5. Mutation
6. 4 or 5
7. AGT
8. Angiotensinogen
9. 7 or 8
10. 3 and 6 and 9

Supplementary material II

Scale for quality assessment of gene polymorphism studies of dilated cardiomyopathy

| Criteria | Score |
| --- | --- |
| Representativeness of cases |  |
| Consecutive/randomly selected from case population with clearly defined sampling frame | 2 |
| Consecutive/randomly selected from case population without clearly defined sampling frame or with extensive inclusion/exclusion criteria | 1 |
| No method of selection described | 0 |
| Representativeness of controls |  |
| Controls were consecutive/randomly drawn from the same sampling frame (ward/community) as cases | 2 |
| Controls were consecutive/randomly drawn from a different sampling frame as cases | 1 |
| Not described | 0 |
| Ascertainment of dilated cardiomyopathy |  |
| Clearly described objective criteria for diagnosis of dilated cardiomyopathy | 2 |
| Diagnosis of dilated cardiomyopathy by patients’ history | 1 |
| Not described | 0 |
| Ascertainment of controls |  |
| Controls were tested to screen out dilated cardiomyopathy, i.e., echocardiography was performed | 2 |
| Controls were subjects who did not report dilated cardiomyopathy; no objective testing | 1 |
| Not described | 0 |
| Genotyping examination |  |
| Genotyping done under ‘‘blinded’’ condition for M235T genotype or DD genotypes samples were confirmed with an insertion specific PCR to exclude mistyping | 1 |
| Unblinded or not mentioned | 0 |
| Hardy-Weinberg equilibrium |  |
| Hardy-Weinberg equilibrium in the control group | 2 |
| Hardy-Weinberg disequilibrium in the control group | 1 |
| No checking for Hardy-Weinberg equilibrium | 0 |
| Confounding bias |  |
| No difference in ethnic origin between cases and controls | 2 |
| Use of controls who were not related to cases/use of genomic controls | 1 |
| No report of what was done | 0 |

Supplementary material IIIa Risk of bias assessment for included studies (*ACE* genotype)

| First Author, Year | Representativeness of cases | Representativeness of controls | Ascertainment  of DCM | Ascertainment  of controls | Genotyping examination | Test of HWE | Confounding  bias | Total score |
| --- | --- | --- | --- | --- | --- | --- | --- | --- |
| Kong 2012 | 2 | 2 | 2 | 1 | 0 | 0 | 2 | 9 |
| Zou 2003 | 2 | 2 | 2 | 1 | 0 | 2 | 1 | 10 |
| Wu 2002 | 2 | 0 | 2 | 0 | 0 | 2 | 0 | 6 |
| Shan 2001 | 2 | 2 | 2 | 1 | 0 | 2 | 1 | 10 |
| Kose 2014 | 2 | 2 | 2 | 1 | 1 | 2 | 1 | 10 |
| Mahjoub 2010 | 2 | 2 | 2 | 1 | 1 | 2 | 2 | 12 |
| Küçükarabaci 2008 | 0 | 0 | 2 | 2 | 0 | 0 | 1 | 5 |
| Rai 2008 | 2 | 2 | 2 | 1 | 0 | 2 | 2 | 11 |
| Jurkovicova 2007 | 2 | 0 | 2 | 1 | 0 | 2 | 1 | 8 |
| Covolo 2003 | 2 | 2 | 1 | 2 | 0 | 2 | 2 | 11 |
| Tiago 2002 | 2 | 2 | 2 | 1 | 0 | 0 | 2 | 9 |
| Tiret 2000 | 2 | 2 | 2 | 1 | 0 | 2 | 2 | 11 |
| Straburzynska-Migaj 2005 | 2 | 1 | 2 | 1 | 0 | 2 | 1 | 9 |
| Candy 1999 | 2 | 2 | 2 | 1 | 0 | 2 | 2 | 11 |
| Vancura 1999 | 2 | 2 | 2 | 1 | 0 | 2 | 1 | 10 |
| Yamada 1997 | 2 | 2 | 2 | 1 | 0 | 2 | 1 | 10 |
| Sanderson 1996 | 2 | 2 | 2 | 1 | 0 | 2 | 1 | 10 |
| Montgomery 1995 | 2 | 2 | 2 | 1 | 0 | 2 | 1 | 10 |
| Raynolds 1993 | 2 | 2 | 2 | 2 | 0 | 0 | 1 | 9 |
| Ozhan 2004 | 2 | 2 | 2 | 1 | 1 | 2 | 1 | 11 |
| Kurbanov 2013 | 0 | 0 | 2 | 2 | 0 | 0 | 0 | 4 |
| Harn 1995 | 2 | 2 | 2 | 2 | 0 | 0 | 1 | 9 |
| Rani 2017 | 2 | 2 | 2 | 1 | 1 | 2 | 2 | 12 |
| Schmidt 1996 | 2 | 2 | 2 | 2 | 0 | 0 | 2 | 10 |
| Chen 2017 | 2 | 2 | 2 | 1 | 0 | 2 | 2 | 11 |
| Goncalvesova 2005 | 2 | 0 | 2 | 0 | 0 | 0 | 0 | 4 |
| Berg 2012 | 2 | 2 | 2 | 1 | 0 | 2 | 2 | 11 |

Note, DCM, dilated cardiomyopathy; HWE, Hardy Weinberg Equilibrium, full scores were 13.

Supplementary material IIIb Risk of bias assessment for included studies (*AGT* genotype)

| First Author, Year | Representativ eness of cases | Representativeness of controls | Ascertainment of DCM | Ascertainment of controls | genotyping examination | Test of HWE | Confounding bias | Total score |
| --- | --- | --- | --- | --- | --- | --- | --- | --- |
| Jurkovicova 2007 | 2 | 0 | 2 | 1 | 0 | 2 | 1 | 8 |
| Tiago 2002 | 2 | 2 | 2 | 1 | 0 | 0 | 2 | 9 |
| Tiret 2000 | 2 | 2 | 2 | 1 | 0 | 2 | 2 | 11 |
| Pávková Goldbergová 2011 | 0 | 0 | 0 | 0 | 1 | 2 | 0 | 3 |
| Yamada 1997 | 2 | 2 | 2 | 1 | 0 | 2 | 1 | 10 |
| Rani 2017 | 2 | 2 | 2 | 1 | 1 | 2 | 2 | 12 |
| Ullah 2019 | 2 | 2 | 2 | 1 | 0 | 0 | 2 | 9 |

Note, DCM, dilated cardiomyopathy; HWE, Hardy Weinberg Equilibrium, full scores were 13.
